# Type 1 Fimbriae, a Colonization Factor of Uropathogenic *Escherichia coli*, Are Controlled by the Metabolic Sensor CRP-cAMP

**DOI:** 10.1371/journal.ppat.1000303

**Published:** 2009-02-20

**Authors:** Claudia M. Müller, Anna Åberg, Jurate Straseviçiene, Levente Emődy, Bernt Eric Uhlin, Carlos Balsalobre

**Affiliations:** 1 Department of Molecular Biology and Laboratory for Molecular Infection Medicine Sweden (MIMS), Umeå University, Umeå, Sweden; 2 Institute of Medical Microbiology and Immunology, University of Pécs Medical School, Budapest, Hungary; 3 Veterinary Research Institute, Hungarian Academy of Sciences, Budapest, Hungary; 4 Departament de Microbiologia, Universitat de Barcelona, Barcelona, Spain; Institut Pasteur, France

## Abstract

Type 1 fimbriae are a crucial factor for the virulence of uropathogenic *Escherichia coli* during the first steps of infection by mediating adhesion to epithelial cells. They are also required for the consequent colonization of the tissues and for invasion of the uroepithelium. Here, we studied the role of the specialized signal transduction system CRP-cAMP in the regulation of type 1 fimbriation. Although initially discovered by regulating carbohydrate metabolism, the CRP-cAMP complex controls a major regulatory network in Gram-negative bacteria, including a broad subset of genes spread into different functional categories of the cell. Our results indicate that CRP-cAMP plays a dual role in type 1 fimbriation, affecting both the phase variation process and *fimA* promoter activity, with an overall repressive outcome on fimbriation. The dissection of the regulatory pathway let us conclude that CRP-cAMP negatively affects FimB-mediated recombination by an indirect mechanism that requires DNA gyrase activity. Moreover, the underlying studies revealed that CRP-cAMP controls the expression of another global regulator in Gram-negative bacteria, the leucine-responsive protein Lrp. CRP-cAMP-mediated repression is limiting the switch from the non-fimbriated to the fimbriated state. Consistently, a drop in the intracellular concentration of cAMP due to altered physiological conditions (e.g. growth in presence of glucose) increases the percentage of fimbriated cells in the bacterial population. We also provide evidence that the repression of type 1 fimbriae by CRP-cAMP occurs during fast growth conditions (logarithmic phase) and is alleviated during slow growth (stationary phase), which is consistent with an involvement of type 1 fimbriae in the adaptation to stress conditions by promoting biofilm growth or entry into host cells. Our work suggests that the metabolic sensor CRP-cAMP plays a role in coupling the expression of type 1 fimbriae to environmental conditions, thereby also affecting subsequent attachment and colonization of host tissues.

## Author Summary

Attachment of bacteria to the surface of host tissues is a crucial initial step in the establishment of bacterial infections. This process is mediated by adhesins, such as the type 1 fimbriae of *Escherichia coli*, which play a key role during urinary tract infections by mediating adhesion to the uroepithelium. The expression of type 1 fimbriae is finely regulated attending to environmental signals and is under phase variation control, which determines the percentage of fimbriated cells in the population. In this report, we show that the expression of type 1 fimbriae is repressed by a metabolic sensor of the cell, the global regulatory complex CRP-cAMP. We demonstrate that CRP-cAMP affects the switching outcome by selectively inhibiting the recombination process in one direction only, resulting in a lower percentage of fimbriated cells. Such a switch to the non-fimbriated state after successful adhesion might be advantageous in the urinary tract, where the immune mechanisms of the host favor the removal of bacteria expressing immunogenic surface structures. Understanding the regulatory networks that govern regulation of virulence and colonization factors is both of basic interest and might help to develop novel strategies to treat bacterial infections.

## Introduction

Bacteria have the ability to rapidly adapt to changes in the environment, a feature that is important for survival and multiplication both during colonization of host organisms and in the environment. An efficient adaptation implies the ability to sense external parameters and to transduce the perceived signals to cellular regulators, which then incite adaptive changes in the physiology of the cell. One form of signal transduction occurs via cytoplasmatic secondary messenger systems, so-called alarmones, which can mediate a rapid response. Alarmones are low molecular mass, non-proteinaceous, enzymatically synthesized compounds. Several modified nucleotides have been described to execute this function in bacteria, among them the 3′,5′-cyclic adenosine monophosphate (cAMP). cAMP is a ubiquitous molecule found in both prokaryotes and eukaryotes. In bacteria, the activity of cAMP was initially thought to be restricted to its role in catabolite repression [1]. However, there is evidence for an extended role of cAMP as sensory signal involved in global gene regulation in bacteria [2]–[5]. The level of intracellular cAMP is modulated by several environmental factors [6]–[8]. The cellular target for cAMP-signaling is the cAMP receptor protein (CRP). Dimeric CRP in complex with one molecule of cAMP exhibits DNA-binding activity to sites located near promoter regions [9]. Thereby, CRP-cAMP acts as a global regulator of gene expression by controlling the expression of almost 200 operons in *E. coli* [10]–[12].

Type 1 fimbriae mediate attachment to both biotic and abiotic surfaces and are involved in the early stages of biofilm formation [13],[14]. In *E. coli*, type 1 fimbriae play a crucial role during urinary tract infections by mediating adhesion to mannose-containing receptors on the uroepithelium and promoting the formation of intracellular bacterial communities [15]–[17]. Those adhesins are encoded by the *fim* determinant composed of two independent transcription units coding for the recombinases FimE and FimB, and a polycistronic operon encoding the structural components (FimA, FimF, FimG, and FimH) and a pilus assembly system (FimC and FimD) [18],[19]. Phase variable expression of the *fim* operon is associated with the inversion of a 314-bp chromosomal region, flanked by two 9-bp inverted repeats, that contains the *fimA* promoter [20],[21]. When the invertible element is in the so-called ON orientation, the promoter is directed towards the structural *fim* genes, thus allowing transcription, whereas transcription is abolished in the inverted OFF orientation. The inversion process is catalyzed by FimB and FimE, two members of the tyrosine site-specific recombinase family [22],[23].

Several regulators are involved in the fine modulation of the expression of type 1 fimbriae by environmental conditions [24],[25]. A proper supercoiling state of the DNA and the presence of accessory proteins, such as the DNA binding proteins Lrp and IHF, are essential features that affect the recombination process and determine whether the cell is fimbriated or not [26]–[29]. Other regulators such as RpoS, ppGpp, NanR and NagC modulate type 1 fimbriation mostly by altering the expression of the recombinases that catalyze the recombination event [30]–[32]. Moreover, the global regulator H-NS has been shown to affect type 1 fimbriation both by regulating the expression of the recombinases and by directly interacting with the *fim* invertible element [33],[34]. Effects on the expression of type 1 fimbriae in *cya* derivatives of *E. coli* K-12, which are defective in cAMP synthesis, were reported earlier [35]. However, different strains responded divergently upon addition of exogenous cAMP in static cultures and it was not clarified at what level the reported effects were operating. In this work, we describe that CRP-cAMP represses type 1 fimbriation. The dissection of the mechanism underlying the observed phenomenon demonstrated that CRP-cAMP indirectly represses FimB-mediated recombination during the phase variation process. In contrast to many other regulators of the phase variation of type 1 fimbriae described, CRP-cAMP affects phase variation independently of the levels of the recombinases. We propose a novel model by which CRP-cAMP controls the type 1 fimbriation state in the bacterial population by affecting DNA gyrase activity. In addition, our studies led to the new discovery that Lrp expression in *E. coli* is under the control of the CRP-cAMP complex.

## Results/Discussion

### Type 1 fimbriation in *E. coli* is enhanced by the lack of the CRP-cAMP regulatory complex

For a successful colonization of hosts by bacteria, it is crucial that the expression of bacterial surface structures, which mediate the interaction with the host tissues, is finely regulated. In *E. coli*, the CRP-cAMP complex has been shown to regulate the production of several of those surface structures, such as flagella or P-fimbriae [36]–[39]. Using a *crp* deletion mutant derivative of the extensively studied uropathogenic *E. coli* (UPEC) isolate J96, we further characterized the role of CRP-cAMP in the modulation of the expression of those colonization factors. Confirming previous data, the CRP-cAMP deficient derivatives were non-motile and had lost the ability to cause mannose-resistant haemagglutination (MRHA) (data not shown). Agglutination tests using specific antisera against the Pap and Prs fimbriae, adhesins that mediate MRHA, confirmed that the expression of those fimbriae is strictly dependent on the presence of functional CRP-cAMP in the cell (data not shown).

J96, as most of the UPEC isolates, also expresses type 1 fimbriae, which are essential for the adherence and invasion of the bladder uroepithelium. The expression of type 1 fimbriae can be detected by mannose-sensitive yeast agglutination (MSYA), which attests the ability of type 1 fimbriated bacteria to bind to mannosides-containing receptors on the surface of yeast cells. A clear stimulation in the ability to cause MSYA was observed in the J96*crp* strain as compared with wt when growing in various culture media (LB, TBA, CFA, and TSA; data not shown). Semi-quantitative MSYA, using serially diluted LB cultures, corroborated these results: agglutination of yeast cells was observed with a higher dilution of the J96*crp* cell suspension (4-fold, i.e. containing 8-times less bacterial cells) as compared to wt (Table 1). These results indicate that the deficiency in CRP-cAMP caused a substantial increase in the expression of type 1 fimbriae on the cell surface.

**Table 1:** Semi-quantitative MSYA in wt and *crp* derivatives of different bacterial strains

		Genotype	
Strain		wt	*crp*
J96	*fim*_J96_	1/2	1/8
VL751/pACYC184	*fim* ^−^	n.d.	n.d.
VL751/pSH2	*fim*_J96_	1/32	1/128
MG1655	*fim*_MG1655_	1/4	1/8

Bacterial cultures of the indicated strains (wt and *crp*) were grown in LB medium overnight at 37°C with vigorous shaking. The origin of the *fim* determinant present in each strain is indicated. The numeric values indicate the highest dilution of the bacterial culture that agglutinated yeast cells. n.d. = no MSYA detected.

In UPEC, a regulatory crosstalk between fimbrial operons occurs, which also affects the expression of type 1 fimbriae. It is known that the UPEC-specific regulators PapB, SfaB, and FocB, which are involved in the regulation of P-related, S-related, and F1C-related fimbriae, respectively, have the ability to repress the expression of type 1 fimbriae [40]–[42]. It has been described that CRP-cAMP is essential for the expression of the operon encoding the P-related fimbriae and the PapB regulator [39]. Therefore, a possible explanation for the observed increase in type 1 fimbriation in the *crp* derivative could be the lack of expression of UPEC-specific repressors of type 1 fimbriae such as PapB. To test this, MSYA experiments were performed using wt and *crp* derivatives of an *E. coli* K-12 strain, which lacks such regulators. The VL751 strain (mutant in the chromosomal *fim* gene cluster and consequently deficient in type 1 fimbriation), carrying the entire *fim* determinant of the J96 strain on the pACYC184-based plasmid pSH2 was used. *crp* derivatives of this commensal strain expressing the *fim*_J96_ determinant had enhanced agglutination ability as compared with the wt strain (Table 1). As expected, strains carrying pACYC184 did not agglutinate yeast cells. Furthermore, type 1 fimbriae expression was also monitored in the K-12 strain MG1655 (*fim* ^+^) and its *crp* derivative, giving the same result (Table 1). As a control, in all MSYA assays described, the agglutination could be effectively blocked by the addition of mannosides (data not shown). Since the up-regulation of type 1 fimbriation was observed in both UPEC and commensal isolates, our results exclude the possibility that the CRP-cAMP effect strictly requires any UPEC-specific factor or is solely due to regulatory crosstalk between fimbrial operons, although we can not rule out a possible contribution to the regulation in pathogenic isolates.

### CRP-cAMP plays a dual role in the transcriptional regulation of type 1 fimbriae

The *fimA* gene encodes the major subunit of the type 1 fimbriae and its expression is phase variable. Transcriptional studies were performed using two lineages derived from AAEC198A and AAEC374A strains, which carry the same *fimA-lacZ* operon fusion in the chromosome. The AAEC374A derivatives CBP374 (wt) and CBP375 (Δ*crp*) are phase variation deficient due to mutations in the *fimB* and *fimE* genes (encoding the site-specific recombinases) and have the invertible element locked in the ON orientation. Such strains were used to monitor the transcriptional activity of the *fimA* promoter. The AAEC198A derivatives CBP198 (wt) and CBP199 (Δ*crp*) are phase variation proficient and consequently, the *fimA* expression monitored using these strains integrates both the percentage of *fim*-expressing cells (phase variation) and the activity of the *fimA* promoter.

**Figure 1:**
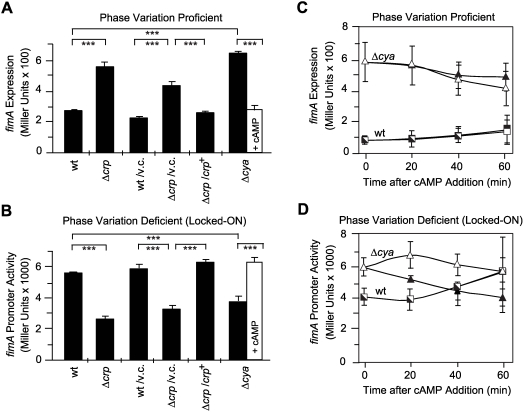
Effect of CRP-cAMP deficiency on the expression of the *fimA* gene. (A–B) *fimA* expression was monitored by measuring ß-galactosidase activity from a chromosomal transcriptional *fimA-lacZ* fusion in either phase variation proficient (A) or phase variation deficient (B) strain backgrounds. For complementation purposes, plasmid pCBP68 (*crp* ^+^) carrying the *crp* gene in the vector pLG338 (v.c.) was used. Bacterial cultures were grown in LB medium at 37°C to mid-log phase. Black bars represent values derived from cultures grown without cAMP and white bars represent values obtained from cultures grown in presence of 5 mM cAMP. Strains used in A: CBP198 (wt), CBP199 (Δ*crp*), and CMM198 (Δ*cya*); Strains used in B: CBP374 (wt), CBP375 (Δ*crp*), and CMM374 (Δ*cya*). (C–D) Effect of addition of 5 mM cAMP (open symbols) on *fimA* expression in either phase variation proficient (C) or phase variation deficient (D) strain backgrounds. Bacterial cultures were grown in LB medium at 37°C to an OD_600nm_ of 0.05 before the addition of cAMP. As controls, cultures with no addition of cAMP (filled symbols) were used. Strains used in C: CBP198 (wt, squares) and CMM198 (Δ*cya*, triangles). Strains used in D: CBP374 (wt, squares) and CMM374 (Δ*cya*, triangles). All results shown are the mean values and standard deviations from three independent experiments.

Using strains CBP198 and CBP199, a clear *fimA* up-regulation was observed in the *crp* background as compared with wt (Fig. 1A), indicating that CRP-cAMP represses type 1 fimbriation at the transcriptional level, consistent with the agglutination data (Table 1). On the other hand, when using CBP374 and CBP375 strains (Fig. 1B), a significantly lower *fimA* promoter activity was detected in the *crp* mutant, thereby suggesting that CRP-cAMP stimulates *fimA* promoter activity itself. Having in consideration that CRP activity is strictly dependent on its co-factor cAMP, in-frame *cya* deletion mutant strains (cAMP-deficient strains) were created and compared with isogenic *crp* and wt strains. The effect of the *cya* mutation on *fimA* expression resembled the one observed in the *crp* strain (Fig. 1A and 1B). Moreover, restoration of CRP-cAMP activity in *crp* and *cya* derivatives by using either the low copy number plasmid pCBP68 (pLG338-*crp*) or external addition of cAMP, respectively, restored *fimA* expression to wt levels (Fig. 1A and 1B). Taken together, we may conclude that CRP-cAMP has a dual role in the transcriptional expression of type 1 fimbriae: i) to stimulate transcription of the *fimA* promoter in phase-ON-cells and, ii) to repress the overall type 1 fimbriae expression.

A response in the expression of type 1 fimbriae upon addition of exogenous cAMP was reported in static cultures of different *cya* derivative strains [35]. However, the response was divergent in different bacterial strains. The physiological heterogeneity in static cultures might be the cause of the strain-dependent variation detected. Our experiments using shaken cultures growing under uniformly aerated conditions gave identical results with all the strains used. Moreover, an increased *fimA* transcriptional expression in a CRP-cAMP deficient strain could be extracted from a microarray dataset on the effect of the *crp* mutation on the global pattern of expression in *E. coli* [10], consistent with our results.

To further probe into the dual role of CRP-cAMP in *fimA* expression, the effect of rapid restoration of CRP activity by addition of cAMP to *cya* strains was studied. When using early log phase cultures of the phase variation proficient strain CBP198 and its *cya* counterpart (Fig. 1C), the exposure to cAMP during one hour period did not significantly alter the expression of *fimA* when compared to the expression in control cultures (no addition of cAMP). However, a significant rapid alteration in expression (*p* = 0.036 after 20 minutes) as a response to the addition of cAMP was detected when using the phase variation deficient strains CBP374 (wt) and CMM374 (Δ*cya*) (Fig. 1D). The differences in the kinetic of the response suggest that the dual role of CRP-cAMP on *fimA* expression might be achieved by distinct mechanisms: a direct stimulation of the *fimA* promoter activity and an indirect role in the overall negative effect of CRP-cAMP on type 1 fimbriae expression.

### CRP-cAMP affects phase variation of type 1 fimbriae

**Figure 2:**
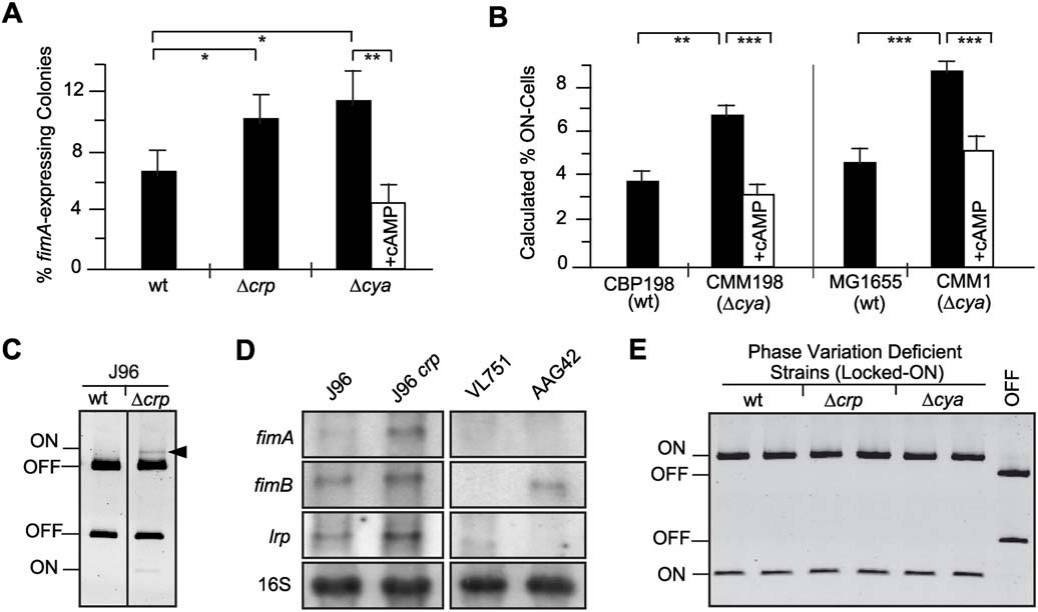
The percentage of fimbriated cells in the population is increased in *crp* and *cya* strains. (A) The percentage of *fimA*-expressing cells in presence (white bar) or absence (black bars) of 5 mM cAMP was determined by the indicator plate assay (see Materials and Methods) using mid-log phase cultures of the strains CBP198 (wt), CBP199 (Δ*crp*) and CMM198 (Δ*cya*). Mean values and standard deviations from three independent experiments are shown. (B) Quantification of the percentage of ON-cells in bacterial populations by a PCR-based assay. Cultures of wt and *cya* derivatives of strains CBP198 and MG1655 were grown to mid-log phase in presence (white bars) or absence (black bars) of 5 mM cAMP. Mean values and standard deviations of three independent experiments are shown. (C) ON-OFF diagnostic of mid-log phase cultures of the J96 strain and its *crp* derivative; the arrowhead highlights the fragment corresponding to ON-cells detected in the J96*crp* samples. (D) Northern hybridization of total RNA extracted from mid-log cultures from strains J96 (wt), J96*crp* (Δ*crp*), VL751 (Δ*fim*), and AAG42 (Δ*lrp*) with specific probes for *fimA*, *fimB*, *lrp,* and 16S rRNA as indicated. (E) ON-OFF diagnostic of duplicated cultures of the phase variation deficient strains CBP374 (wt), CBP375 (Δ*crp*), and CMM374 (Δ*cya*). A control showing the band pattern of an OFF population was included for comparison. The pictures in panels C and E are electronically inverted images of ethidium bromide stained acrylamide gels.

So far, we have described that CRP-cAMP deficiency causes: i) a higher degree of type 1 fimbriation, ii) an increase in the expression of type 1 fimbriae in phase variation proficient strains, and iii) a reduction in *fimA* promoter activity in phase variation deficient strains. These results suggest that the percentage of fimbriated cells (ON-cells) in both *crp* and *cya* mutant strains is elevated when compared to wt, causing the overall increase in *fimA* expression. To test this prediction, the percentage of ON-cells in the population was monitored by plating cultures of the phase variable *fimA-lacZ* reporter strains on indicator plates containing X-gal. As predicted, a significant increase in the percentage of ON-cells in both the *crp* and *cya* mutant strains was observed (Fig. 2A). A quantitative PCR based method was validated (see Materials and Methods and Fig. S1) and used to detect and quantify the subpopulations having the invertible element either in the ON or in the OFF orientation [40]. Consistent with the results obtained from indicator plates, a significantly higher percentage of ON-cells was found for CMM198 (Δ*cya*) as compared to CBP198 (wt) (Fig. 2B). Moreover, the *cya* deficiency was chemically complemented by addition of exogenous cAMP in the culture medium. The effect of CRP-cAMP deficiency in the switching between the ON and OFF orientation was further corroborated when *in vivo* switching frequencies were measured (see below). Similar results were obtained when using the reporterless and type 1 fimbriation-proficient strain MG1655 and its *cya* mutant derivative (Fig. 2B), thereby excluding the possibility that the *lacZYA* DNA sequence present in the reporter strains might affect the CRP-mediated effect on phase variation. Moreover, when derivatives of the uropathogenic isolate J96 were used, a significant increase in the percentage of ON-cells was detected in *crp* derivatives. While most of the cells in the wt population were in the OFF orientation under the culture conditions used, a subpopulation of cells with the invertible element in the ON orientation was clearly detected in the mutant derivative (Fig. 2C). To confirm these results, the level of *fimA* transcript in cultures of J96 and its derivative J96*crp* were quantified by Northern blot analysis (Fig. 2D). A 2.4-fold increase in *fimA* transcript was detected in J96*crp* as compared with wt, corroborating the results obtained when using *fimA*-*lacZ* reporter strains (Fig. 1A).

The drop in *fimA* expression in CRP-cAMP deficient strains carrying mutations in *fimB fimE* (Fig. 1B) was assumed to indicate a stimulatory effect of CRP-cAMP on *fimA* promoter activity. However, it could also be a consequence of an alteration of the percentage of ON-cells by the action of some alternative FimB/FimE-like recombinase, as described for several *E. coli* strains [43]. As depicted in Fig. 2E, OFF-cells were not observed in cultures of strains CBP374 (wt), CBP375 (Δ*crp*) and CMM374 (Δ*cya*), thereby ruling out the involvement of alternative recombinases, which is consistent with the fact that no genes for such enzymes are detected in the MG1655 genome [43]. Taken together, our results suggest that CRP-cAMP acts on the phase variation process by causing a decrease in the percentage of fimbriated cells in the population.

### Physiological implications of the CRP-cAMP–mediated regulation of type 1 fimbriation

**Figure 3:**
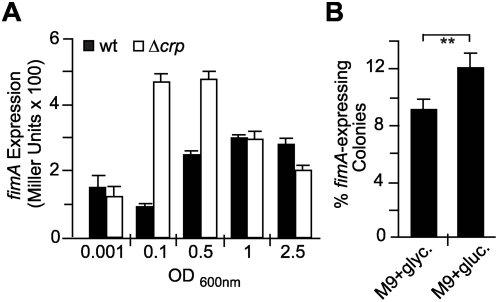
Type 1 fimbriae expression profile in different growth conditions. (A) *fimA* expression was determined by measuring ß-galactosidase activity at various optical densities from cultures of the *fimA-lacZ* reporter strains CBP198 (wt, black bars) and CBP199 (Δ*crp*, white bars) in LB medium at 37°C. (B) Quantification of the percentage of *fimA*-expressing cells in the population of strain CBP198 (wt) on indicator plates. Cultures were grown to mid-log phase at 37°C in M9 minimal medium containing either glycerol (glyc.) or glucose (gluc.) as a carbon source. Mean values and standard deviations from three independent experiments are shown.

Type 1 fimbriation is growth phase dependent [31],[32]. The *fimA* expression profile throughout the growth curve was studied using the strains CBP189 (wt) and CPB199 (Δ*crp*). As previously described, *fimA* expression in wt cultures was low in the early growth stages, increased in the middle of the logarithmic phase, and stayed constantly high throughout stationary phase (Fig. 3A). In contrast, *fimA* expression in the *crp* mutant peaked during early logarithmic phase and then dropped down to almost wt levels during late-logarithmic phase. Consistent with the transcriptional data, a larger difference in the semi-quantitative phenotypic determination of type 1 fimbriae expression (MSYA) was observed with mid-log phase cultures of the wt and *crp* derivatives of MG1655 (1/2 versus 1/8, respectively) when compared to stationary phase cultures (1/4 versus 1/8, Table 1). The analysis of *fimA* expression through the growth curve suggests that CRP-cAMP represses type 1 fimbriation in actively growing cells, while during growth arrest, the repression is released and other global regulators such as RpoS and ppGpp assume the control [31],[32]. This finding is also in agreement with the described growth phase-dependent levels of CRP-cAMP in the cell. As assessed by Northern blot analysis, *crp* transcriptional expression is high in early exponential phase and significantly reduced in stationary phase [44].

A well-described factor that alters the intracellular levels of CRP-cAMP is carbon source availability, e.g. the presence of glucose causes a significant reduction [6],[7]. The effect of glucose on the expression of type 1 fimbriae was monitored. A modest but significant increase in the percentage of *fimA*-expressing cells could be observed when CBP198 (wt) cultures were grown in M9-glucose medium compared with cultures grown in M9-glycerol (Fig. 3B). The stimulatory effect of the presence of glucose on transcriptional expression of type 1 fimbriae was also observed by microarray analysis on the effect of glucose in the general expression pattern in *E. coli* [45].

CRP-cAMP deficient strains have a significant growth defect compared to the wt (i.e: 89 and 34 minutes generation time in LB for CBP199 and CBP198, respectively), which might raise the question whether the increased type 1 expression in the *crp* strains is merely due to the growth alterations. However, growth in media that significantly increases the growth rate of the *crp* strain, i.e. LB medium containing glucose (32 and 48 minutes generation time for CBP198 and CBP199, respectively), did not alter the difference in the expression of type 1 fimbriae between the wt and the *crp* strains (data not shown), suggesting that the CRP specific effect on type 1 fimbriae expression is not coupled to the growth rate.

### CRP-cAMP affects the FimB-mediated OFF to ON switch both *in vivo* and *in vitro*

**Figure 4:**
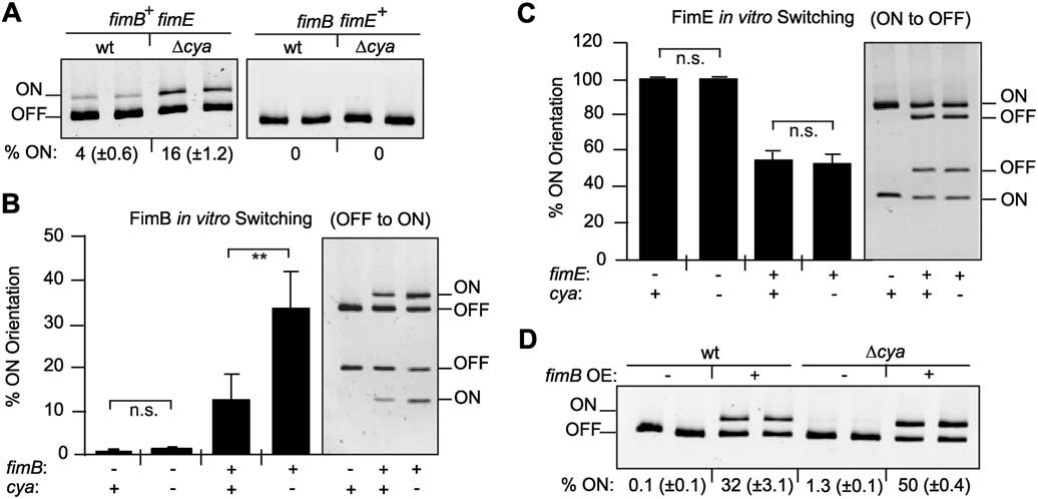
The CRP-cAMP regulatory complex affects the FimB-mediated OFF to ON switch. (A) Determination of the percentage of ON-cells in strains expressing either FimB (AAEC370A, *fimB* ^+^ *fimE*) or FimE (AAEC261A, *fimB fimE* ^+^) and their *cya* counterparts. The upper half of a representative gel used for ON-OFF diagnostic is shown. The estimated percentage of ON-cells in the cultures (% ON) is indicated as mean values and standard deviations in brackets of three independent experiments. (B) *In vitro* OFF to ON recombination assay in bacterial extracts either containing FimB or being recombinase free. Bacterial extracts were obtained from strain NEC026 (*fim*, *cya* ^+^) and its isogenic *cya* mutant CMM026, transformed with either an inducible *fimB* expression plasmid (pIB378, *fimB*+) or the vector control (pET11, *fimB*−). Extracts were mixed with the template plasmid pJL-2 (*fim* invertible element in the OFF orientation). The orientation of the plasmid-encoded *fim* invertible element was determined after 3 h incubation by using the PCR-based assay (see Materials and Methods). Results are provided in the bar diagram as percentage of invertible elements in the ON orientation. The picture in the right part of the Figure illustrates an ethidium bromide stained gel from one of the experiments used to obtain the data shown. (C) *In vitro* ON to OFF recombination assay in bacterial extracts either containing FimE or being recombinase free. A similar assay as presented in B was performed. In this case, bacterial extracts were obtained from strain NEC026 (wt) or its isogenic *cya* mutant CMM026, transformed with either an inducible *fimE* expression plasmid (pIB382, *fimE*+) or the vector control (pET11, *fimE*−). Extracts were mixed with the template plasmid pMM36 (*fim* invertible element in the ON orientation) and analyzed as in B. In both B and C, mean values and standard deviations from four independent experiments are shown. (D) Effect of CRP-cAMP deficiency in the percentage of ON-cells in cultures of strain J96 and its *cya* derivative carrying either the plasmid pPKL9 (constitutive *fimB* expression, *fimB* OE+) or the vector control (pBR322, *fimB* OE−). The percentage of ON-cells was quantified from cultures of two independent clones. In A and D, the image corresponds to the upper half of an ethidium bromide stained gel.

The reported increase in the percentage of ON-cells in the CRP-cAMP deficient strains could be achieved either by stimulating the OFF to ON inversion (exclusively catalyzed by FimB) or by causing the opposite effect on the ON to OFF inversion (mainly catalyzed by FimE). To further dissect the role of CRP-cAMP in the recombination event, the percentage of ON-cells in wt and *cya* derivative strains expressing either FimB (AAEC370A, *fimE*) or FimE (AAEC261A, *fimB*) was determined (Fig. 4A). In the FimB proficient strains (*fimE*), a significant increase in the percentage of ON-cells was detected in the strain lacking CRP-cAMP (16% in *cya* versus 4% in wt). However, in FimE proficient strains (*fimB*), consistent with published results [46], all cells were in the OFF orientation independently of the presence or absence of the CRP-cAMP complex. These results suggest that CRP-cAMP is directly or indirectly affecting the FimB-mediated inversion. To corroborate these data, *in vitro* recombination assays were performed using template plasmids as recombination substrate in bacterial extracts of *cya* and *cya* ^+^ strains overexpressing either FimB or FimE. The induction of the synthesis of the recombinases in cultures of the *cya* and *cya* ^+^ strains provided apparently identical amounts of the enzymes in the extracts of both strains as determined by Coomassie-stained SDS-PAGE (Fig. S2). When FimB-mediated OFF to ON inversion was monitored (Fig. 4B), recombination occurred with both *cya* and wt extracts in the presence of FimB. However, a remarkable 3-fold higher percentage (*p* = 0.003) of invertible fragments in the ON orientation was detected in the extract from the *cya* strain when compared with wt extracts. On the other hand, FimE-mediated inversion from the ON to the OFF state did not seem to be affected by a mutation in the *cya* gene (Fig. 4C). The FimB recombinase can also catalyze the switch from ON to OFF. However, no effect of CRP-cAMP on the FimB-mediated ON to OFF inversion was detected when *in vitro* recombination assays with DNA template in the ON orientation were performed (data not shown). Altogether, our *in vitro* studies corroborate the results obtained *in vivo* and suggest that the CRP-cAMP complex specifically affects the FimB-mediated recombination event from the OFF to the ON orientation. Supporting this conclusion, *in vivo* switching frequency estimations indicated that the OFF to ON switching rate was significantly increased in strain CBP199 (Δ*crp*) as compared with CBP198 (wt) (1.1×10^−4^ and 1.4×10^−2^ per cell and generation in wt and mutant, respectively), while no significant effect was observed in the ON to OFF switching (1.0×10^−6^ and 1.6×10^−6^ per cell and generation in wt and mutant, respectively). Also supporting our results, it was reported that the FimB-mediated switching frequency from OFF to ON is 3-fold higher in the presence of glucose (i.e. reduced intracellular levels of CRP-cAMP) than in the presence of glycerol [24].

Although higher expression of *fimB* was observed in *crp* derivatives as compared to wt counterparts in both J96 and MG1655 strains (Fig. 2D and data not shown), the *in vitro* data, where the recombinases were overexpressed to the same degree in both extracts, suggest that the enhanced OFF to ON switching in absence of CRP-cAMP is not strictly dependent on the levels of the FimB recombinase. To further test this hypothesis, *in vivo* experiments were performed under conditions of constitutive *fimB* expression using plasmid pPKL9, which contains the *fimB* gene under the control of the *tet* promoter (Fig. 4E). Control experiments by Northern blot analyses verified that the *fimB* expression levels from plasmid pPKL9 were essentially identical in CRP-cAMP proficient and deficient genetic backgrounds (data not shown). The percentage of ON-cells in cultures of J96 derivatives constitutively expressing *fimB* (carrying plasmid pPKL9) was significantly elevated in the CRP-cAMP deficient strain as compared with wt, yielding a 50% higher percentage of ON-cells. Comparable results were obtained when using MG1655 derivative strains (data not shown). Altogether, our results both *in vivo* and *in vitro* indicate that the CRP-cAMP complex has a negative effect on the switching process independently of the intracellular concentration of the FimB recombinase.

### CRP-cAMP represses type 1 fimbriation by an indirect mechanism

Two possible mechanisms by which CRP-cAMP affects the FimB-mediated switch should be considered: either CRP-cAMP can directly interact with the invertible DNA fragment repressing the OFF to ON switch, or the effect of CRP-cAMP may be indirect.

**Figure 5:**
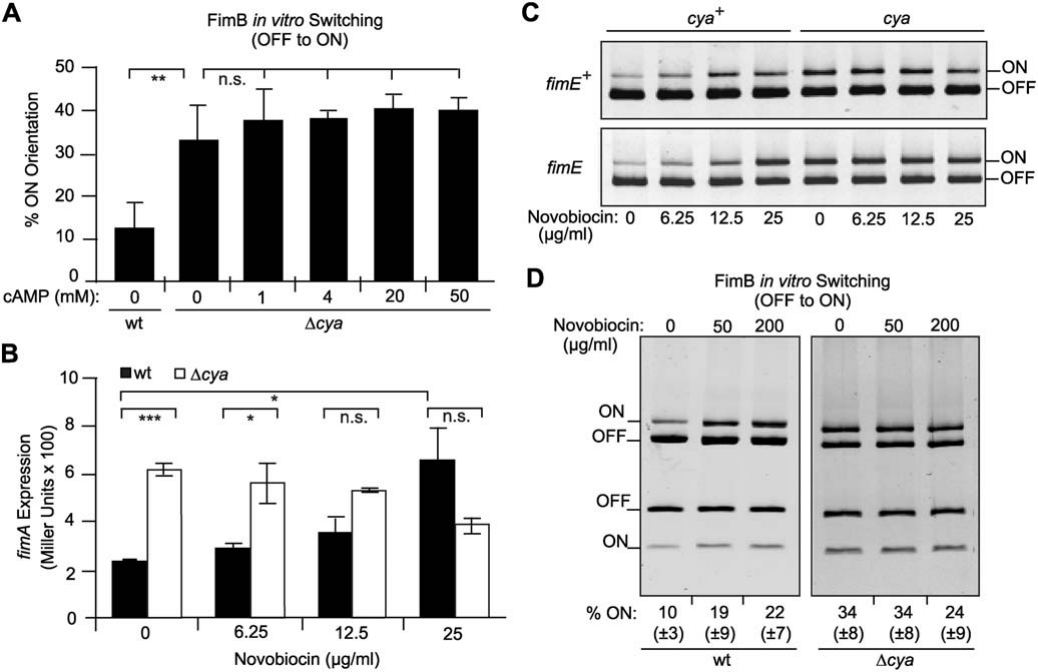
Insights on the mechanism of action of the CRP-cAMP complex *in vivo* and *in vitro*. (A) Effect of the addition of cAMP during *in vitro* OFF to ON recombination. Bacterial extracts were obtained from strains NEC026 (wt) or CMM026 (Δ*cya*) transformed with the inducible *fimB* expression plasmid (pIB378). Extracts were mixed with the template plasmid pJL-2 in absence or presence of increasing amounts of cAMP (1 to 50 mM final concentration). Mean values and standard deviations of three independent experiments are shown. (B) Effect of increasing amounts of the gyrase inhibitor novobiocin on *fimA* expression. ß-galactosidase activity was measured from strains CBP198 (wt, black bars) and CMM198 (Δc*ya*, white bars) grown to mid-log phase in LB medium supplemented with 0, 6.25, 12.5, and 25 µg ml^−1^ novobiocin. Mean values and standard deviations from two independent experiments are shown. (C) Effect of DNA gyrase inhibition on the orientation of the *fim* invertible element *in vivo*. Upper panel: ON-OFF diagnostic of the samples used in Fig. 5B, representing the strain CBP198 (*fimB* ^+^ *fimE* ^+^) and its *cya* derivative CMM198 grown in presence of novobiocin (concentrations as indicated). Lower panel: ON-OFF diagnostic of the strain AAEC370A (*fimB* ^+^ *fimE*) and its Δ*cya* derivative CMM370A subject to the same growth conditions as in the upper panel. Both panels depict electronically inverted images of the upper half of acrylamide gels after ethidium bromide staining. (D) Effect of DNA gyrase inhibition on FimB-mediated OFF to ON switching *in vitro*. Increasing amounts of novobiocin (0, 50, 200 µg ml^−1^) were added to the *in vitro* recombination reactions. Bacterial extracts from strains NEC026 (wt, left panel) or CMM026 (Δ*cya*, right panel) transformed with the inducible *fimB* expression plasmid (pIB378) were used together with the template plasmid pJL-2. Mean values and standard deviations in brackets of the estimated percentage of invertible elements in the ON orientation from four independent experiments are given as numbers below each lane. The images correspond to ethidium bromide stained gels from a representative experiment used to obtain the data shown.

The slow response when adding exogenous cAMP to CMM198 cultures (Fig. 1C) suggested that the role of CRP-cAMP in the regulation of the phase variation occurs by an indirect mechanism. Nevertheless, to establish whether CRP-cAMP might also be directly involved in the switching process, *in vitro* recombination assays were performed using extracts of the *cya* strain while restoring CRP-cAMP activity by addition of increasing amounts of cAMP (Fig. 5A). No obvious alteration in the FimB-mediated switch was detected, strongly suggesting that CRP-cAMP does not directly interact with the nucleoprotein complex that is the substrate for the FimB recombinase. Accordingly, no effect was observed in the outcome of *in vitro* recombination assays when purified CRP was added to extracts obtained from a *crp* strain (data not shown).

Simultaneously, the possible binding of CRP-cAMP to various DNA fragments spanning different regions of the *fim* determinant was tested (Fig. S3A). No strong CRP binding was detected to any of the DNA fragments tested. At most, a low affinity binding was detected in case of the fragment containing the *fimA* promoter (PCR7; Fig. S3B). However, when DNase I footprinting analysis of this putative CRP binding site was performed, no binding was observed (data not shown). It has been reported that CRP-cAMP might bind to many low affinity binding sites along the *E. coli* chromosome [47]. Although it is possible that such low affinity CRP binding site(s) may exist in the *fimA* promoter region, our experimental evidence (Fig. 5A) suggested that binding is not required for the phase variation control. A possible involvement of the putative CRP binding site(s) in the positive control of the *fimA* promoter activity (Fig. 1B) will be further studied.

### Inhibition of DNA gyrase activity mimics the effect of CRP-cAMP on type 1 fimbrial phase variation

Recently, it has been shown that inhibiting the DNA gyrase promotes the FimB-mediated inversion from OFF to ON and therefore it was concluded that DNA supercoiling determines the directionality of the FimB-mediated recombination [29],[48]. DNA gyrase is an enzyme that catalyses ATP-dependent DNA breakage, strand passage and rejoining of double-stranded DNA (for a recent review see Nöllmann *et al*. [49]). DNA gyrase is involved in the regulation of DNA topology, but also in other processes such as replication or illegitimate recombination [50],[51]. Remarkably, it has been described that CRP-cAMP modulates the expression of the *gyrA* gene encoding the DNA gyrase. In *crp* deficient strains, low levels of *gyrA* expression and DNA gyrase activity, monitored as alterations in the topology of plasmid DNA, were detected [52]. One may hypothesize that the CRP-cAMP mediated effect on the FimB-recombination process could directly result from the low levels of DNA gyrase activity detected in *crp* deficient strains. To test this hypothesis, the effect of inhibiting the DNA gyrase *in vivo* was analyzed in both wt (CBP198) and *cya* (CMM198) strains (Fig. 5B). Addition of increasing amounts of novobiocin (DNA gyrase inhibitor) in wt cultures caused a concomitant increase in *fimA* expression, consistent with previously reported data [29],[48]. Remarkably, the *fimA* expression level was essentially unaltered by addition of novobiocin in cultures of the *cya* mutant strain. In agreement with the hypothesis proposed, the novobiocin mediated inhibition of the DNA gyrase caused an increase in the percentage of ON-cells in the wt strain, but not in the *cya* derivative (Fig. 5C, upper panel). Results that further corroborated our hypothesis were obtained by inducing overexpression of DNA gyrase from cloned *gyrAB* genes in the *cya* strain CMM198. Both repression of *fimA* expression and reduction in the percentage of ON-cells were detected (Fig. S4). Moreover, when *fimE* mutant derivatives were used, thus only reflecting FimB-mediated inversion, an identical response to novobiocin was observed, indicating that the recombination process that was responsive to gyrase inhibition *in vivo* is FimB-specific (Fig. 5C, lower panel). To rule out the possibility that the *lacZYA* sequences present in the *fimA*-*lacZYA* fusion might cause alterations in the regional DNA supercoiling and consequently affect the phase variation, similar experiments were performed using reporterless derivatives of strains MG1655 and J96. Similar results were obtained: i) an increase in the percentage of ON-cells in the wt strains was observed after addition of increasing novobiocin concentration (5-fold and 2-fold increase with the highest concentration of novobiocin tested in MG1655 and J96 strains, respectively), ii) the level of ON-cells was not altered by novobiocin treatment in the CRP-cAMP deficient derivatives, and iii) the percentage of ON-cells in the wt achieved by novobiocin treatment was similar to the level detected in the CRP-cAMP deficient derivatives (data no shown). It is noteworthy that in all approaches (Fig. 5B and 5C) the presence of novobiocin in sub-inhibitory concentrations did not significantly alter the expression of *fimA* in the *cya* mutant strains, which is in agreement with a low DNA gyrase activity in the CRP-cAMP deficient background as a result of the low expression of the *gyrA* gene [52].

To corroborate the *in vivo* results obtained, *in vitro* analyses were performed where increasing amounts of novobiocin were added to the wt strain extract. A progressive increase in the OFF to ON switching efficiency *in vitro* was observed (Fig. 5D), consistent with our *in vivo* data and with previously reported data [29],[48]. Moreover, the unaltered *fimA* expression in the *cya* mutant strain by addition of novobiocin, together with the *in vitro* switching data, indicates that the *fimA* promoter is indifferent to changes in the DNA gyrase activity, in agreement with previous data [48].

The fact that inhibition of the DNA gyrase activity *in vitro* stimulated the FimB-mediated recombination suggests an active role of the DNA gyrase during the recombination process itself. Altogether, our data provide evidence that the induction of type 1 fimbriation detected in the CRP-cAMP deficient strains is a process mediated by the alteration in DNA gyrase activity and therefore can be mimicked by the specific inhibition of this enzymatic activity by novobiocin. Moreover, our *in vitro* results comparing OFF to ON switching between wt and *cya* mutant using the same DNA template for both extracts (Fig. 4B and Fig. 5D) demonstrated that the CRP-cAMP effect on phase variation is not merely dependent on the initial supercoiling state of the *fim* invertible element.

### Lack of CRP-cAMP results in increased Lrp levels

**Figure 6:**
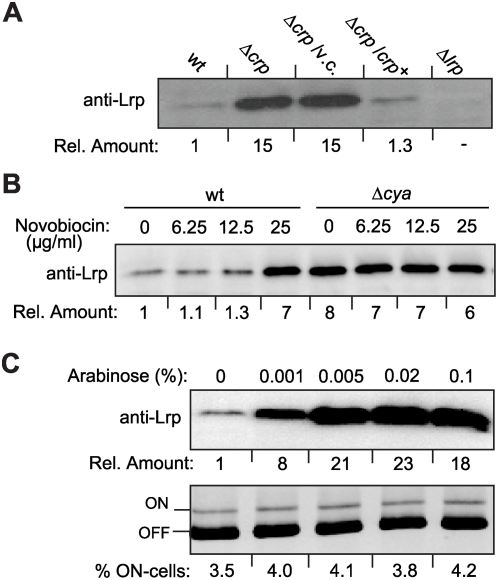
Induction of Lrp in a CRP-cAMP deficient background. (A) Lrp levels in different genetic backgrounds. Whole bacterial cell lysates of the strains CBP198 (wt), CBP199 (Δ*crp*), CBP199/pLG339 (Δ*crp*/v.c.), CBP199/pLG339-CRP (Δ*crp*/*crp*), and AAG42 (Δ*lrp*) were subjected to immunoblot analyses using Lrp-specific antiserum. Numbers below each lane represent the average of the signal intensity from three independent experiments relative to the corresponding wt value (set as one). (B) Analysis of Lrp levels in whole cell lysates of strain CBP198 (wt) and CMM198 (Δ*cya*) grown in the presence of novobiocin at the indicated concentrations (same bacterial cultures as in Fig. 5B). (C) Effect of overexpression of *lrp* on the percentage of ON-cells. Cultures of strain CBP198 transformed with a plasmid that carries the *lrp* gene under the inducible P_ara_-promoter (pAAG6) were grown to mid-log phase in presence of the indicated concentrations of arabinose. The induced levels of *lrp* were assessed by immunoblotting using Lrp-specific antiserum. Simultaneously, quantification of the percentage of ON-cells in the populations was performed by using the PCR-based method. The lower image corresponds to the upper half of a representative gel used for ON-OFF diagnostic; the results are given as numbers below each lane.

Recombination at the *fim* invertible element requires Lrp, a DNA bending protein that directly binds to specific sites within the invertible element and stimulates DNA inversion [26]. Kelly *et al.* [29] demonstrated that this binding activity of Lrp is required to promote the FimB-mediated OFF to ON directionality observed when the DNA gyrase was inhibited. The Lrp levels were determined by immunoblot analysis of *crp* ^+^ and *crp* strains (Fig. 6A) and the Lrp content detected was several fold higher in the *crp* strains than in the *crp* ^+^ strains. These results suggest a possible link between the CRP and Lrp regulons. Interestingly, a direct demonstration of CRP dependent regulation of Lrp expression has not been done, although two putative CRP sites have been predicted in the promoter region of the *lrp* gene [53], suggesting a possible direct regulation by CRP-cAMP. Additionally, CRP-cAMP could act indirectly by positively regulating GadE, which represses *lrp* expression [54],[55]. Transcriptional studies have been performed by Northern blot analysis of RNA from derivatives of MG1655 and J96 (Fig. 2D). An increase in the level of the *lrp* transcript in the *crp* derivatives was detected as compared with wt (2.5 and 1.7- fold in MG1655 and J96 derivatives, respectively), suggesting a role for CRP-cAMP in the control of *lrp* transcription, although an additional regulation at the posttranscriptional level can not be ruled out. Further studies would be required to characterize the CRP-cAMP dependent regulation of Lrp expression.

Interestingly, when the levels of intracellular Lrp were monitored in the same cultures as in Fig. 5B, again a differential effect of the inhibition of the DNA gyrase in wt and *cya* strains was observed (Fig. 6B). In a wt strain, the Lrp levels were strongly elevated at the highest concentration of novobiocin, where the amount of Lrp was apparently identical to the amount detected in the *cya* strain in absence of novobiocin, which again might be explained by the low DNA gyrase activity detected in the CRP-cAMP deficient background [52]. We tested whether increased levels of Lrp by itself would cause an alteration in the FimB-mediated switching process. Our results clearly indicate that Lrp overexpression *per se* did not result in any significant changes in the percentage of ON-cells when no alteration in DNA gyrase activity was induced (Fig. 6C).

### Concluding remarks

The expression of type 1 fimbriae implies the allocation of an important part of the asset of the bacterial cell for the production of those proteinaceous appendages, as considerable amounts of energy and amino acids are needed for their synthesis. A tightly regulated expression of such organelles is therefore expected. Considering the important metabolic effort performed by the bacterial cells committed to be fimbriated, regulation by phase variation can be seen as a selective advantage for the bacterial population, in addition of providing phenotypical heterogeneity in an otherwise genetically homogeneous population. In previous works, we have shown that the expression of type 1 fimbriae is stimulated when intracellular levels of the stringent response alarmone ppGpp are raised [32],[56]. The level of ppGpp in the cell increases under amino acid starvation and energy stress [57]. In this report, the role of CRP-cAMP, a regulatory complex that is associated with the energy state of the cell, has been included in the extensive list of regulatory networks controlling type 1 fimbriation.

**Figure 7:**
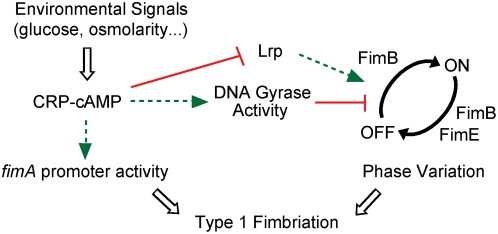
Schematic model of action of the CRP-cAMP complex on the regulation of type 1 fimbriation. The integration of different environmental signals modify the levels of the CRP-cAMP complex which affects the phase variation of type 1 fimbriae by altering the FimB-mediated directionality of the OFF to ON recombination event. Based on our results, a model is proposed where a stimulation of the DNA gyrase activity by CRP-cAMP [52] would repress the FimB-mediated recombination from the OFF to the ON orientation by a mechanism that requires the presence of Lrp [29]. In this work, a repressing effect of CRP-cAMP on the expression of Lrp and a stimulatory effect of CRP-cAMP on *fimA* promoter activity have also been described. Green arrows indicate stimulatory effects, whereas red lines indicate repressing effects.

Interestingly, many of the global regulators that affect type 1 fimbriae expression, such as H-NS, RpoS, Lrp, and now CRP-cAMP, have been shown to interplay among each other, thereby orchestrating gene regulation cascades in response to the growth conditions [58]. From our studies, we can conclude that CRP-cAMP is a major regulator of fimbriation during the exponential growth phase (Fig. 3A) and is required to maintain the growth expression profile of type 1 fimbriae. We dissected the initial observation that the *crp* derivatives of J96 showed an increased ability to agglutinate yeast cells and conclude that CRP-cAMP represses type 1 fimbriation, as schematically shown in Fig. 7, by the recently described mechanism of switching directionality established by the activity of the DNA gyrase and the presence of Lrp [29], thereby affecting FimB-mediated OFF to ON switching. Remarkably, CRP-cAMP inhibited FimB-mediated recombination at a template plasmid isolated from a *crp* ^+^ background, indicating that the regulatory effect does not merely depend on the supercoiling state of the DNA and thereby suggesting an active role of the DNA gyrase in the OFF to ON recombination event. Interestingly, CRP-cAMP has a dual effect on type 1 fimbriation by repressing phase variation and promoting promoter activity. Further studies will be required for fully understanding the underlying mechanisms by which CRP-cAMP affects both levels of regulation of type 1 fimbriation.

In *Salmonella*, *crp cya* mutants are avirulent in a mouse model [59] and it has been reported that the *crp* and the *cya* genes are strongly repressed during infection of macrophages [60]. Moreover, it has been observed that DNA becomes more relaxed when bacteria are growing in certain intracellular environments and consequently the expression of those genes that are required for intracellular survival is induced [61]. Therefore, CRP-cAMP might be involved in controlling *Salmonella* virulence in a pathway that includes DNA supercoiling and the sensing of environmental conditions as previously proposed [62].

It is well described that CRP and cAMP levels are affected by environmental conditions such as glucose availability and osmolarity [6],[7]. Interestingly, such environmental conditions also affect DNA topology in *E. coli* in a DNA gyrase dependent manner [63],[64]. The link between CRP-cAMP mediated regulation of gene expression and DNA gyrase activity might represent a specialized signal transduction pathway that senses the metabolic and energetic status of the cell. It can not be ruled out that in this regulatory pathway others factors such as the FIS protein might be involved. FIS has been proposed earlier as a metabolic sensor involved in the homeostatic control of DNA supercoiling. Interestingly, CRP-cAMP modulates *fis* expression [65]. Supporting this model, a correlation between the sensitivity to catabolite repression and to gyrase inhibitors has been observed earlier for different metabolic operons. Hence, inhibition of DNA gyrase activity represses the expression of several CRP-cAMP sensitive genes (three maltose operons, the lactose and galactose operons, and the tryptophanase gene), whereas CRP-cAMP independent genes such as threonine and tryptophan operons were insensitive to DNA gyrase inhibitors [66],[67]. We can consider a scenario where disadvantageous environmental conditions, which are sensed by the bacterial cells as low rate of energy flow in the cell, are transduced by different pathways (CRP-cAMP regulon, ppGpp regulon), inducing survival strategies including cell fimbriation. The fact that the highly energy-consuming process of fimbriation is stimulated in conditions of starvation highlights the putative selective advantage represented by the ability of expressing type 1 adhesins, which promotes important virulence properties such as biofilm formation and colonization of host mucosa.

The physiological relevance of the CRP-cAMP-mediated signaling pathway controlling type 1 fimbriation needs to be further explored. Nevertheless, the modulation of type 1 fimbriation mediated by glucose availability may provide a possible explanation to the reported observation that diabetic patients commonly are highly susceptible to urinary tract infections [68]. The presence of an unusual high concentration of glucosides in the urine of those patients may cause a reduction in the intracellular level of CRP-cAMP in the bacterial cell. This could result in a concomitant increase in type 1 fimbriation which, in turn, would raise the probability of colonization of the urinary tract.

## Materials and Methods

### Bacterial strains, plasmids, and growth conditions

All strains and plasmids used in this study are listed in Table 2. Strains were grown to mid-log phase (corresponding to an OD_600nm_ of around 0.5) with vigorous shaking (200 rpm) at 37°C in Luria Bertani (LB) medium [69] or in M9 medium [70] supplemented with either 0.4% glycerol or 0.4% glucose, unless otherwise stated. For mannose-resistant haemagglutination (MRHA), bacteria were grown in CFA (1% casaminoacids, 0.15% yeast extract, 0.05% MgSO_4_, 0.005% MnCl_2_). For mannose-sensitive yeast agglutination (MSYA), bacteria were grown in different culture media: TBA (1% tryptone, 0.5% NaCl, 1.5% agar), TSA (1.5% trypticase peptone, 0.5% phytone peptone, 0.5% NaCl, 1.5% agar) and CFA. When necessary, the following antibiotics were used: tetracycline (12.5 µg ml^−1^), carbenicillin (50 µg ml^−1^), kanamycin (25 µg ml^−1^) and chloramphenicol (15 µg ml^−1^). When indicated, cyclic AMP was added in a final concentration of 5****mM. To study type 1 fimbriae expression, cultures of the different strains were always inoculated using colonies showing an OFF (non-fimbriated) phenotype. When *fimA-lacZ* fusion derivatives were used, OFF-colonies could be identified on X-gal plates (white colonies). In reporterless strains (MG1655 and J96 derivatives), the fimbriation state of the inoculum was estimated from the colony morphology, since ON-colonies are small and convex, while OFF-colonies are large and flat, as described by Blomfield *et al.* [71].

**Table 2:** Strains and plasmids used in this study

	Relevant Phenotype	Reference or Source
*Strains*		
J96	Clinical isolate	[81]
BEU805	J96 Δ*crp39*, Tc^r^	This study
CMM96	J96 Δ*cya*::Km^r^	This study
MG1655	λ^−^ F^−^ *fim* ^+^	[82]
BEU742	M182 Δ*crp39*, Tc^r^	[7]
SS5357	Δ*lrp*::Tc^r^	S.A. Short
AAEC198A	MG1655 *ΔlacZYA fimA-lacZYA*	[26]
AAEC374A	MG1655 *ΔlacZYA fimA-lacZYA fimB-am6 fimE-am18*, phase locked ON	[26]
AAG42	AAEC198A Δ*lrp*:: Tc^r^	This study
CBP198	AAEC198A, Tc^r^	This study
CBP199	AAEC198A Δ*crp*39, Tc^r^	This study
CMM198	CBP198 Δ*cya*, Tc^r^	This study
CBP374	AAEC374A, Tc^r^	This study
CBP375	AAEC374A Δ*crp39*, Tc^r^	This study
CMM374	CBP374 Δ*cya*, Tc^r^	This study
AAEC261A	MG1655 Δ*lacZYA fimB-lacZYA*	[26]
CMM261A	AAEC261A Δ*cya*::Km^r^	This study
AAEC370A	MG1655 Δ*lacZYA fimA-lacZYA fimE-am18*	[26]
CMM370A	AAEC370A Δ*cya*::Km^r^	This study
CMM1	MG1655 Δ*cya*	This study
CMM2	MG1655 Δ*cya*::Km^r^	This study
NEC026	BL21 (DE3) Δ*fimB-H*	[79]
CMM026	NEC026 Δ*cya*::Km^r^	This study
VL751	CSH50 ara Δ*(lac-pro) rpsL thi Δfim*	[21]
CBP751	VL751 Δ*crp39*, Tc^r^	This study
*Plasmids*		
pLG338	cloning vector, Tc^r^, Km^r^	[83]
pLG339	cloning vector, Tc^r^, Km^r^	[83]
pACYC184	cloning vector, Tc^r^, Cm^r^	[84]
pBR322	cloning vector, Cb^r^, Tc^r^	[85]
pBAD30	expression vector, P_ara_ promoter, Cb^r^	[86]
pSH2	*fim*-cluster from J96 cloned into pACYC184, Cm^r^	[19]
pCBP68	wt *crp* allele cloned into pLG338, Km^r^	[7]
pLG339-CRP	wt *crp* allele cloned into pLG339, Km^r^	[87]
pET11	T7 promoter expression vector, Cb^r^	[88]
pIB378	*fimB* under T7 promoter cloned into pET11	[79]
pIB382	*fimE* under T7 promoter cloned into pET11	[79]
pMM36	*fim* invertible element cloned into pACYC184 derivative, Cm^r^ (switch ON)	[89]
pJL-2	*fim* invertible element cloned into pACYC184 derivative, Cm^r^ (switch OFF)	[89]
pPKL9	*fimB* under *tet* promoter cloned into pBR322, Cb^r^	[18]
pAAG6	*lrp* under ara promoter cloned into pBAD30, Cb^r^	This study
pCA24N-*gyrA*	*gyrA* under T5-lac promoter cloned into pCA24N, Cm^r^	[90]
pRSFDuet-*gyrB*	*gyrB* under T7-*lac* promoter cloned into pRSFDuet-1, Km^r^	[90]

### Genetic techniques

Standard molecular manipulations were performed according to Sambrook and Russel [70]. The *cya* deletion mutant (Δ21–259) and Δ*cya*::Km^r^ deletion mutant (Δ21–259::Km^r^) were created by allelic exchange as described by Link *et al.* [72]. The deletion mutant and Km^r^ deletion mutant were verified by PCR amplification using primers cya-A and cya-D, and cya-up and cya-D, respectively. Gene alleles were introduced by phage P1-mediated transduction [73] using the following donor strains; BEU742 for Δ*crp39* (Tc^r^), SS5357 for Δ*lrp*::Tc^r^ and CMM2 for Δ*cya*::Km^r^. Derivatives *crp* ^+^ and Δ*crp39* were initially selected by colony size and confirmed by PCR using primers CRP1 and CRP3. The plasmid pAAG6 was constructed by cloning a PCR-amplified fragment spanning the *lrp* gene between the EcoRI-SmaI sites of pBAD30. The PCR fragment was generated using the primers lrp-1 and 64 and MG1655 as template. All primers used are specified in Table S1.

### Mannose-resistant haemagglutination (MRHA)

Bacteria were grown on CFA agar plates. Bacterial cell suspension in PBS containing 3% (w/v) mannose (methyl α-D-mannopyranoside, Sigma) were prepared and adjusted to an OD_600 nm_ of 5. MRHA was tested using suspensions from human (agglutination-positive with P-fimbriae) and dog (agglutination-positive with Prs-fimbriae) erythrocytes (8%, v/v) in PBS, giving identical results. The erythrocytes and bacterial suspensions were mixed in proportion 1:1 (v/v) on a glass-slide. Presence of aggregates was considered as agglutination positive.

### Fimbrial antisera agglutination

Bacteria were grown on CFA agar plates. Bacterial colonies were mixed with 10 µl of 20× diluted antisera. Two types of antisera were used, pPAP5 and pPAP60 (originally obtained against P and Prs fimbriae, respectively) that strongly cross-react and mediate agglutination through both types of fimbriae [74]. Presence of aggregates was considered as agglutination positive.

### Mannose-sensitive yeast agglutination (MSYA)

Bacteria were grown on LB plates overnight at 37°C, washed in PBS and resuspended to an OD_600nm_ of 5. Yeast cells (*Saccharomyces cerevisiae*) were washed and resuspended in PBS to an OD_600nm_ of 5. The suspensions were mixed in a 1:1 (v/v) ratio on a glass-slide placed on ice. After 30 min, the presence of aggregates as sign for agglutination was assessed and scored as + (weak) or ++ (strong). Semi-quantitative analysis was performed as described before [32].

### Motility assay

Overnight cultures were diluted in LB and adjusted to an OD_600nm_ of 1.0 and 4 µl were put on semi-solid LB medium containing 0.3% agar and incubated at 37°C.

### Measurement of β-galactosidase activity

The β-galactosidase assay was performed as described by Miller [75]. Data presented represent average values and standard deviations of duplicate measurements from at least three independent experiments.

### Determination of the percentage of *fimA*-expressing cells on indicator plates

To monitor the orientation of the *fim* invertible element in strains carrying a chromosomal *fimA-lacZ* fusion, indicator plates containing X-Gal (5-bromo-4-chloro-3-indolyl-ß-D-galactopyranoside) were used as previously described [24]. Cells having the *fim* invertible element in the ON orientation give rise to blue colonies, whereas those cells having it in the OFF orientation exhibit white color.

### Determination of the switch orientation and quantification of the percentage of ON-cells using a PCR based assay

The orientation of the invertible DNA fragment can be determined using a molecular approach described previously [32],[40]. In brief, a 602-bp DNA fragment containing the *fim* invertible element was PCR amplified using primers 2535 and 3137, *Hin*fI restricted and analyzed on TBE-acrylamide-gels. Depending on the orientation of the *fim* invertible element, this method generates different sized fragments (484 and 118 bp when in the ON orientation, 402 and 200 bp when in the OFF orientation).

Quantification of the percentage of ON-cells in a specific sample was performed as described by Aberg *et al.* [32]. To prove the reliability of this method, calibration experiments were performed in triplicate using templates from strain CBP374 (a 100% ON-cells sample) and CBP198 (representing the OFF-cells samples). Both strains were grown to the same optical density and mixed such that the fraction of ON-cells in the template varied from 0 to 100%. PCR amplification, *Hin*fI digestion and gel electrophoresis were performed as described above. The applicability of this method to quantify the percentage of ON-cells in bacterial populations was evidenced by a linear correlation [regression coefficient (R^2^) of 0.998] between the percentage of ON-cells in the sample and the intensity of the bands (Fig. S1). Since the invertible element in CBP198 is not completely in the OFF orientation, the intensity derived from ON-cells in the “100%-OFF” sample was subtracted from all intensity values.

### RNA isolation and Northern analysis

Total RNA was extracted from mid-log cultures using the hot-phenol method [76]. Contaminating DNA was removed by DNase I (Roche Diagnostics) treatment for 1 h at 37°C, followed by RNA cleanup using the RNeasy Mini kit (Qiagen). For Northern analysis, 20 µg of total RNA were separated on a formaldehyde:agarose gel and transferred to Hybond-N membranes (Amersham) by capillary blotting as described [77]. The membrane was hybridized with ^32^P-labeled DNA-fragments corresponding to the coding sequences of *fimA, fimB*, *lrp*, and *rrnA*, which were PCR generated using the primer pairs fimA-RT1&2, fimB-RT1&2, lrp-RT1&2 and 16S-RT1&2, respectively. After hybridization overnight at 52°C, membranes were subsequently washed in 1× SSC-0.1% SDS for 15 min at room temperature and in 0.1× SSC-0.1% SDS for 15 min at 52°C. Autoradiograms were obtained using StoragePhosphor screens (Molecular Dynamics), which were scanned using the Storm Imaging System (Molecular Dynamics).

### Determination of FimB and FimE switching frequencies *in vivo*

FimB and FimE-promoted switching frequencies were measured as previously described [24].

### *In vitro* recombination assay

To perform FimB *in vitro* recombination assays, bacterial extracts were obtained from cultures of the *fim* mutant strain NEC026 and its isogenic *cya* mutant strain harboring the plasmid pIB378 (*fimB* gene under the control of an IPTG inducible promoter in pET11). *fimB* expression was induced with 0.4 mM IPTG after the cultures grown in minimal MOPS [78] at 28°C had reached an OD_600nm_ of 0.15. Cells were harvested after 24 h of induction at 28°C and processed as described [40],[79]. As control, extracts lacking FimB were obtained from cultures of the same strains carrying the pET11 plasmid. To perform FimE *in vitro* recombination assays, bacterial extracts were obtained from cultures of the strain NEC026 and its isogenic *cya* mutant strain transformed with the plasmid pIB382 (*fimE* gene under the control of an IPTG inducible promoter in pET11). Cultures were manipulated as described above. The *in vitro* recombination assay was performed as described [40],[79]. The resulting orientation of the invertible element was analyzed after 3 h incubation at 37°C using the PCR-based method described above.

### Immunoblotting

Whole cell extracts from bacterial cultures were separated by SDS-PAGE as described by Laemmli [80] using 15% polyacrylamide gels. Samples were transferred to PVDF membranes using a semidry blotting apparatus. After blocking the membranes overnight in Tris-buffered saline containing 0.1% Tween-20 (TBS-T) and 5% skimmed milk, membranes were incubated for 1 h at room temperature with 2,000× diluted Lrp-specific antiserum or 6,000× diluted PapA-specific antiserum [38] in TBS-T containing 5% skimmed milk. After 3× 15 min washes in TBS-T, membranes were incubated for 1 h with 20,000× diluted anti-rabbit immunoglobuline-horseradish peroxidase conjugate (Dianova, Hamburg, Germany). After further washing, membranes were developed using the enhanced chemiluminescence (ECL+) kit (GE Healthcare) and analyzed on a Chemidoc System (BioRad) equipped with the QuantityOne® Software for quantification.

### Statistical analysis

Differences between average values were tested for significance by performing an unpaired, two-sided Student's t-test. The levels of significance of the resulting *p* values are reported by the following symbols: * = *p*<0.05; ** = *p*<0.01; *** = *p*<0.001 and n.s. = non-significant.

## Supporting Information

Figure S1 Validation of the PCR based assay used for quantifying the percentage of cells in the population with the invertible element in the ON-orientation. Suspensions of CBP198 (OFF-template) and CBP374 (ON-template) cells were mixed in the indicated ratios (0–100% ON-template). The *Hin*fI restriction pattern of the PCR amplified fragment containing the *fim* invertible element is shown. The size (in base pairs) of the different diagnostic ON and OFF fragments is indicated. Below each lane, the results of quantification and calculation of the percentage of ON-cells in the population in each sample according to the described method are presented as mean values±standard deviation of three independent calibration experiments.
(0.32 MB TIF)

Figure S2 Electrophoretic analysis of protein extracts used for the *in vitro* recombination assays. Extracts from NEC026 (wt) and CMM026 (*cya*) strains carrying the plasmids pET11 (vector control), pIB378 (pET11 carrying the *fimB* gene) and pIB382 (pET11 carrying *fimE*) were obtained as described in materials and methods. Proteins were separated by SDS-15% PAGE and Coomassie stained. The bands representing the induced FimB and FimE (right margin) and the molecular mass of relevant protein markers (left margin) are indicated.
(0.44 MB TIF)

Figure S3 DNA-binding pattern of CRP at the *fim* regulatory regions. A. Schematic representation of the *fim* determinant and position of the promoters (P) of *fimB*, *fimE*, and *fimA*. Black arrowheads represent the inverted repeats flanking the invertible element. The relative positions of the PCR fragments used for the gel mobility shift are depicted below. B. Gel mobility shift assay of purified CRP protein (3 µM) and various PCR-amplified DNA fragments (PCR1-5, and PCR7, here shown using OFF-cells as template for amplification). In all panels, samples correspond to: lane 1: no protein (cAMP present); lane 2: CRP protein with 20 mM cAMP (active form); lane 3: CRP protein without cAMP (inactive form). Identical result was obtained when PCR7 fragment was obtained from ON-cells as template. Recombinant CRP protein was purified from strain pp6/pHA7 essentially as described (Zhang *et al*., 1991). The PCR amplification and the mobility shift assay were performed as described (Xia *et al*., 2000). - Zhang, X.P., Gunasekera, A., Ebright, Y.W., and Ebright, R.H. (1991) J. Biomol. Struct. Dyn. 9: 463-473. - Xia, Y., Gally, D., Forsman-Semb, K., and Uhlin, B.E. (2000) EMBO J. 19: 1450-1457
(0.41 MB TIF)

Figure S4 Effect of *gyrA* and *gyrB* overexpression on the expression of type 1 fimbriae in a CRP-cAMP deficient strain. A. *fimA* expression from strain CMM198 (*cya*) carrying the plasmids pCA24N-*gyrA* and pRSFDuet-*gyrB*, which carry the *gyrA* and the *gyrB* genes, respectively, under IPTG-inducible promoters, was monitored in either the absence (−) or the presence (+) of IPTG. Bacterial cultures were grown in LB medium to mid-log phase. IPTG was added at a final concentration of 0.015 mM (condition that did not cause any deleterious effect on the bacterial growth). Mean values and standard deviations from three independent experiments are shown. B. ON-OFF diagnostic of two of the bacterial cultures used in A. The panel depicts an electronically inverted image of the upper half of acrylamide gel after ethidium bromide staining; the arrowhead highlights the fragment corresponding to ON-cells.
(0.10 MB TIF)

Table S1 Oligonucleotides used in this study.
(0.03 MB DOC)
