# Supplementary figures and images for: Type 1 Fimbriae, a Colonization Factor of Uropathogenic Escherichia coli, Are Controlled by the Metabolic Sensor CRP-cAMP
Source: PLoS Pathog. 2009 Feb 20;5(2):e1000303. doi: 10.1371/journal.ppat.1000303 (PMC2636892; doi:10.1371/journal.ppat.1000303)

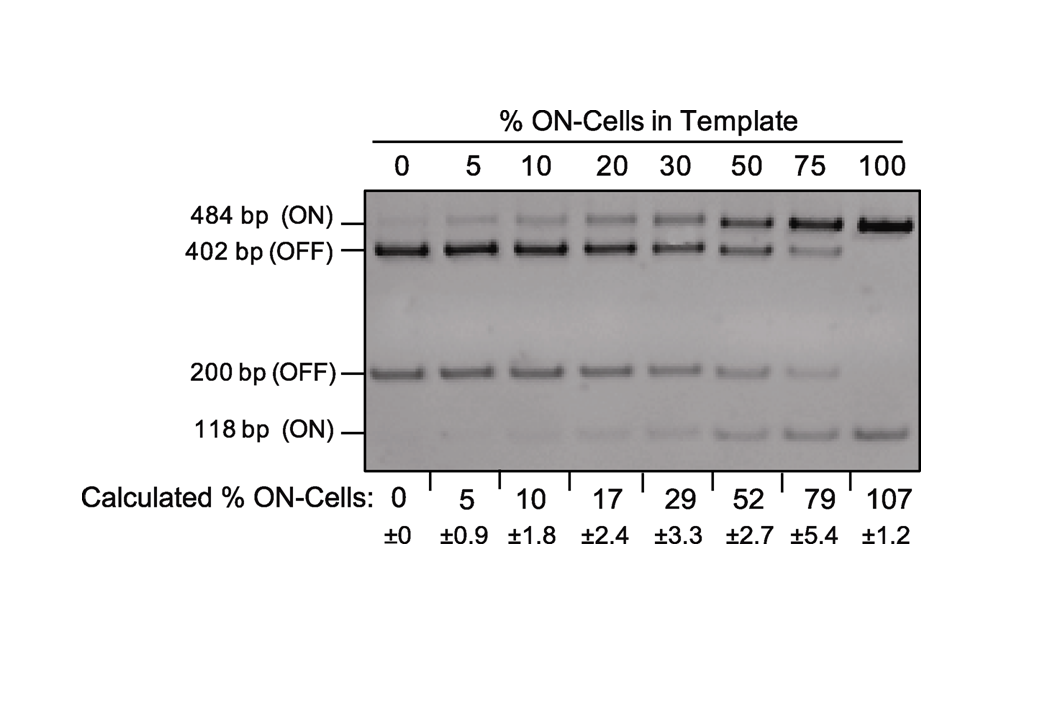

Supplement: Figure S1 — Validation of the PCR based assay used for quantifying the percentage of cells in the population with the invertible element in the ON-orientation. Suspensions of CBP198 (OFF-template) and CBP374 (ON-template) cells were mixed in the indicated ratios (0–100% ON-template). The HinfI restriction pattern of the PCR amplified fragment containing the fim invertible element is shown. The size (in base pairs) of the different diagnostic ON and OFF fragments is indicated. Below each lane, the results of quantification and calculation of the percentage of ON-cells in the population in each sample according to the described method are presented as mean values±standard deviation of three independent calibration experiments. (0.32 MB TIF) [file ppat.1000303.s001.tif]

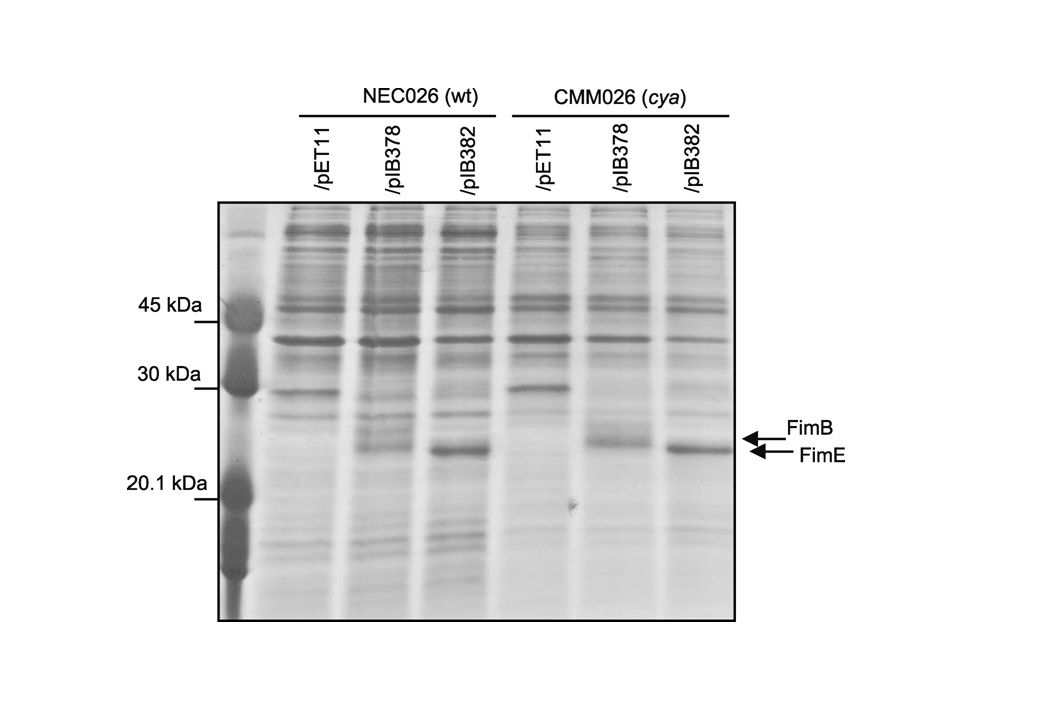

Supplement: Figure S2 — Electrophoretic analysis of protein extracts used for the in vitro recombination assays. Extracts from NEC026 (wt) and CMM026 (cya) strains carrying the plasmids pET11 (vector control), pIB378 (pET11 carrying the fimB gene) and pIB382 (pET11 carrying fimE) were obtained as described in materials and methods. Proteins were separated by SDS-15% PAGE and Coomassie stained. The bands representing the induced FimB and FimE (right margin) and the molecular mass of relevant protein markers (left margin) are indicated. (0.44 MB TIF) [file ppat.1000303.s002.tif]

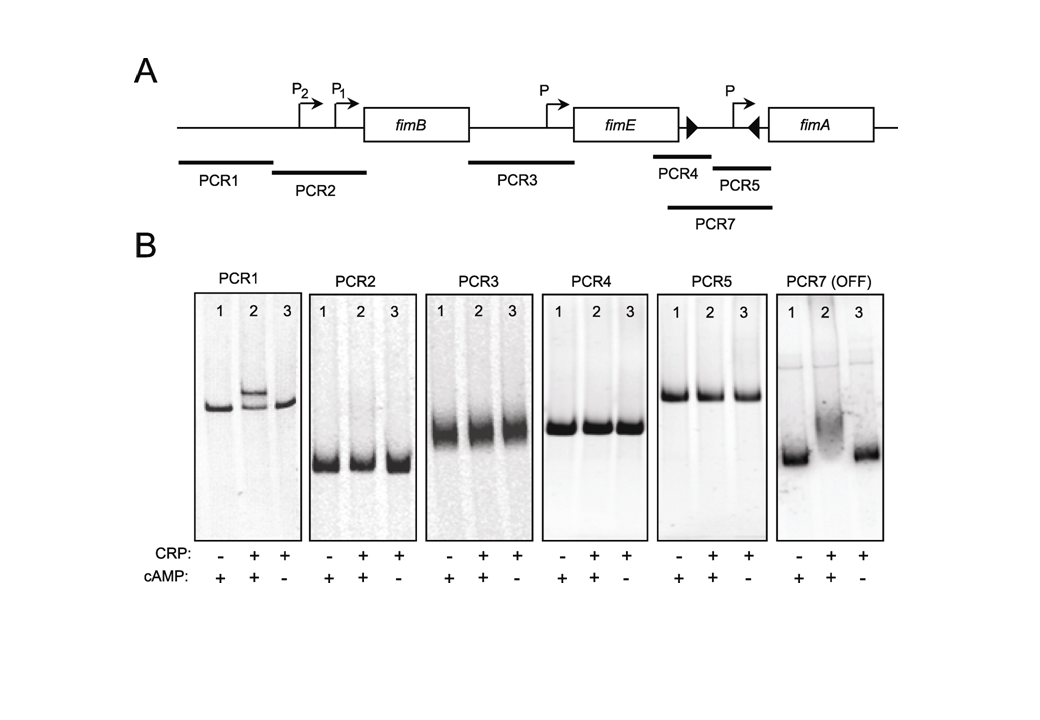

Supplement: Figure S3 — DNA-binding pattern of CRP at the fim regulatory regions. A. Schematic representation of the fim determinant and position of the promoters (P) of fimB, fimE, and fimA. Black arrowheads represent the inverted repeats flanking the invertible element. The relative positions of the PCR fragments used for the gel mobility shift are depicted below. B. Gel mobility shift assay of purified CRP protein (3 µM) and various PCR-amplified DNA fragments (PCR1-5, and PCR7, here shown using OFF-cells as template for amplification). In all panels, samples correspond to: lane 1: no protein (cAMP present); lane 2: CRP protein with 20 mM cAMP (active form); lane 3: CRP protein without cAMP (inactive form). Identical result was obtained when PCR7 fragment was obtained from ON-cells as template. Recombinant CRP protein was purified from strain pp6/pHA7 essentially as described (Zhang et al., 1991). The PCR amplification and the mobility shift assay were performed as described (Xia et al., 2000). - Zhang, X.P., Gunasekera, A., Ebright, Y.W., and Ebright, R.H. (1991) J. Biomol. Struct. Dyn. 9: 463-473. - Xia, Y., Gally, D., Forsman-Semb, K., and Uhlin, B.E. (2000) EMBO J. 19: 1450-1457 (0.41 MB TIF) [file ppat.1000303.s003.tif]

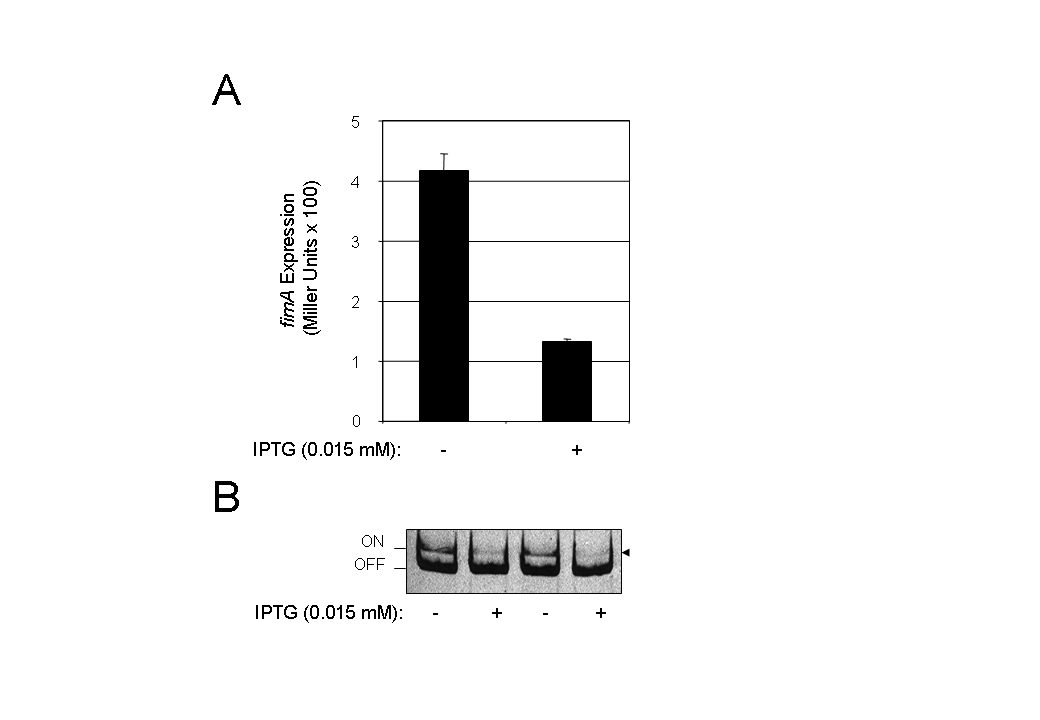

Supplement: Figure S4 — Effect of gyrA and gyrB overexpression on the expression of type 1 fimbriae in a CRP-cAMP deficient strain. A. fimA expression from strain CMM198 (cya) carrying the plasmids pCA24N-gyrA and pRSFDuet-gyrB, which carry the gyrA and the gyrB genes, respectively, under IPTG-inducible promoters, was monitored in either the absence (−) or the presence (+) of IPTG. Bacterial cultures were grown in LB medium to mid-log phase. IPTG was added at a final concentration of 0.015 mM (condition that did not cause any deleterious effect on the bacterial growth). Mean values and standard deviations from three independent experiments are shown. B. ON-OFF diagnostic of two of the bacterial cultures used in A. The panel depicts an electronically inverted image of the upper half of acrylamide gel after ethidium bromide staining; the arrowhead highlights the fragment corresponding to ON-cells. (0.10 MB TIF) [file ppat.1000303.s004.tif]
