# Supplementary material for: Type 1 Fimbriae, a Colonization Factor of Uropathogenic Escherichia coli, Are Controlled by the Metabolic Sensor CRP-cAMP
Source: PLoS Pathog. 2009 Feb 20;5(2):e1000303. doi: 10.1371/journal.ppat.1000303 (PMC2636892; doi:10.1371/journal.ppat.1000303)
Supplement: Table S1 — Oligonucleotides used in this study. (0.03 MB DOC) [file ppat.1000303.s005.doc]

TABLE S1: Oligonucleotides used in this study

| Name | Oligonucleotide Sequence (5’ – 3’) |
| --- | --- |
| 2535 | GCC GGA TTA TGG GAA AGA |
| 3137 | GCC GCT GTA GAA CTG AGG G |
| CRP1 | CAG TCG CGC TTG CAT TTT TGC |
| CRP3 | CCA GGT AAC GCG CCA CTC CG |
| CYA-A | GCC GCG GCC GCC ACA AAT AGT GAC CAG TCC C |
| CYA-D | GCC GTC GAC CGT ATA ACG CTT ACT CGT GGG |
| CYA-UP | TCC GTG GTC CAT CCT AAC ATC C |
| LRP-1 | CCG ATT TCA CGT GAT GTT TCA GGG TCA G |
| 64 | TTA GCG CGT CTT AAT AAC CAG ACG |
| fimA-RT1 | CGC TTG CGC AGT TGA TGC AG |
| fimA-RT2 | CGC GCC TGG AAC GGA ATG G |
| fimB-RT1 | GCA GCA AAT ACC GGG CCT C |
| fimB-RT2 | CAA CCC GGC ATT ACC ACC GG |
| lrp-RT1 | GGA CTT TCC CCA ACG CCG TG |
| lrp-RT2 | CCG TGT GTC ATT GAC GCC AG |
| 16S-RT1 | TGC GAA AGC GTG GGG AGC AC |
| 16S-RT2 | GGT TGC GCT CGT TGC GGG AC |
